# A novel alignment-free method for detection of lateral genetic transfer based on TF-IDF

**DOI:** 10.1038/srep30308

**Published:** 2016-07-25

**Authors:** Yingnan Cong, Yao-ban Chan, Mark A. Ragan

**Affiliations:** 1 Institute for Molecular Bioscience and ARC Centre of Excellence in Bioinformatics, The University of Queensland, St Lucia, Brisbane, QLD 4072, Australia; 2 School of Mathematics and Statistics, The University of Melbourne, Parkville, Melbourne, VIC 3010, Australia

## Abstract

Lateral genetic transfer (LGT) plays an important role in the evolution of microbes. Existing computational methods for detecting genomic regions of putative lateral origin scale poorly to large data. Here, we propose a novel method based on TF-IDF (Term Frequency-Inverse Document Frequency) statistics to detect not only regions of lateral origin, but also their origin and direction of transfer, in sets of hierarchically structured nucleotide or protein sequences. This approach is based on the frequency distributions of *k*-mers in the sequences. If a set of contiguous *k*-mers appears sufficiently more frequently in another phyletic group than in its own, we infer that they have been transferred from the first group to the second. We performed rigorous tests of TF-IDF using simulated and empirical datasets. With the simulated data, we tested our method under different parameter settings for sequence length, substitution rate between and within groups and post-LGT, deletion rate, length of transferred region and *k* size, and found that we can detect LGT events with high precision and recall. Our method performs better than an established method, ALFY, which has high recall but low precision. Our method is efficient, with runtime increasing approximately linearly with sequence length.

Many microbes can acquire DNA from their environment and incorporate it into their genome *via* processes of lateral genetic transfer (LGT; also known as horizontal gene transfer, HGT)^1^. Circumstantial evidence for LGT was first reported more than a century ago^2^, and the phenomenon gained widespread attention in the 1950s with the emergence and spread of multi-drug resistance in bacteria^3^. With the uptake of genome sequencing over the last two decades, it has become increasingly clear that LGT plays a central role in the evolution of microbial genomes^1^,^4^,^5^,^6^. LGT not only contributes to the spread of antibiotic resistance, but is also responsible for a range of metabolic innovations involving carbon and nitrogen metabolism, ion transport and other core processes^7^, which in turn can define microbial physiology and thus ecosystem function.

The recognised mechanisms of LGT (transformation, transduction and conjugation) can introduce exogenous regions of very different lengths, from short fragments to large chromosomal blocks^8^. Recombination need not be constrained by gene boundaries^9^, and there is little evidence to suggest that entire genes, or structurally based regions within genes, are privileged units of transfer^10^,^11^. In any event, genomic regions of lateral origin can be overwritten, wholly or in part, by subsequent LGT events. Thus microbial genomes can become mosaics, with regions of different lengths reflecting the history of LGT events, transfer mechanisms and donors in each lineage. Further, over time, sequence regions of lateral origin will evolve to become indistinguishable from the non-lateral background, a process known as amelioration^12^.

This complex biology presents challenges for the detection and delineation of genomic regions of lateral origin. As typically applied, approaches based on the topological comparison of inferred phylogenetic trees implicitly take genes (gene families) as the unit of analysis. Extensions that test for recombination breakpoints are computationally intensive, yet fail to identify the specific lineage(s) affected by transfer and/or subsequent overwriting. Directionality of transfer can also be difficult or impossible to determine by any phylogenetic approach. More broadly, computational methods are differentially sensitive to the extent of amelioration^13^,^14^. Considerable scope thus remains for the development of new methods that are sensitive, directional, scalable, informative on individual genomes or lineages, and do not require the units of analysis to be delineated *a priori*.

Alignment-free approaches to detect LGT at genome level have been developed in recent years. ALFY (ALignment-Free local homologY)^15^,^16^ uses *Kr*^17^ based on *shustrings* (SHortest Unique subSTRINGS) to calculate pairwise evolutionary distances between genomes, which can then serve as input into a neighbor-joining algorithm^18^ to compute a phylogenetic tree. Then ALFY compares the generated tree with a reference, inferring topological incongruence as instances of LGT.

Another alignment-free method for LGT detection is based on the so-called purity measure^19^. This is a concept from text mining, and is used to detect unusual regions of a string without recourse to domain knowledge. If most substrings of string *x*, which is itself a substring of string *T*, appear with the same frequency as *x*, then the purity value of *x* is high, *i.e.* subpatterns in *x* occur infrequently in *T* outside whole occurrences of *x*, as would be expected if *x* had arisen by LGT. Both of these alignment-free methods use suffix trees^20^ for scalability on large sequence datasets. However, they consider only one target sequence (although ALFY incorporates a pairwise comparison between query and multiple subject sequences) and do not take into account any natural group structure of the dataset, whether taxonomic (a hierarchy of species, genera etc.), ecological or otherwise.

In this paper, we propose a novel alignment-free method for LGT detection based on concepts from TF-IDF (Term Frequency-Inverse Document Frequency). TF-IDF is a numerical statistic from document analysis that reflects the importance of a word (term) to a document within a collection or corpus, by comparing the frequency of a word in a document with its occurrence in other documents.

Term frequency (TF) is used to indicate the topic of a document^21^. The TF of term *t* in document *D* is simply the raw frequency of *t* in *D*, denoted by *tf(t, D)*. The inverse document frequency (IDF)^22^ is used to distinguish a word from the prevalent vocabulary in the corpus. If *t* appears in *D*_*t*_ articles, then its IDF is *idf(t)* = D^*^/*D*_*t*_, where D^*^is the number of all documents in the corpus. Thus a high IDF indicates that the term appears infrequently, and as such carries more importance for a specific article. Salton and Buckley combined the TF and IDF statistics into a single statistic that is widely used as a weighting factor in text mining and information retrieval^22^,^23^,^24^.

Here we apply concepts from TF-IDF to develop an algorithm to detect LGT events in microbial genomes. Using simulated datasets, we test this algorithm and compare its performance with ALFY on sets of sequences of different length, from the size of a single gene (1000 nucleotides) up to 300-fold longer, and evaluate its performance over *k*-mer length and a biologically relevant range of values for parameters including substitution rate between groups, within groups and post-LGT. We find that with appropriate parameter values, the algorithm performs with good precision and recall; furthermore, runtime increases approximately linearly with sequence length, and in most cases TF-IDF performs much better than ALFY^15^. We also apply this method to an empirical dataset composed of seven *Staphylococcus aureus* genomes, and recover putative regions of lateral origin that correspond to genes involved in transport, antibiotic resistance, pathogenicity and virulence. Our results are comparable with those found with ALFY, and include two genomic regions independently confirmed by Holden *et al*.^25^.

## Results

### Performance with different parameter values

**Figure 1:**
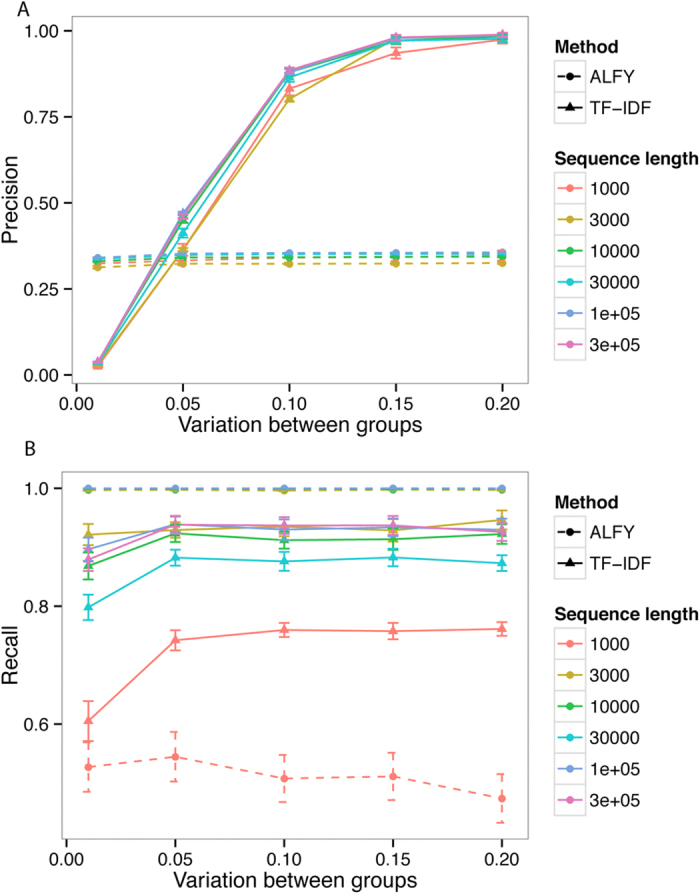
Performance of TF-IDF with variation between groups. Precision (**A**) increases with variation between groups. Recall (**B**) is not substantially affected by variation between groups. Variation within groups is 0.01, variation post-LGT is zero, and deletion is zero. Error bars are 2× standard error.

**Figure 2:**
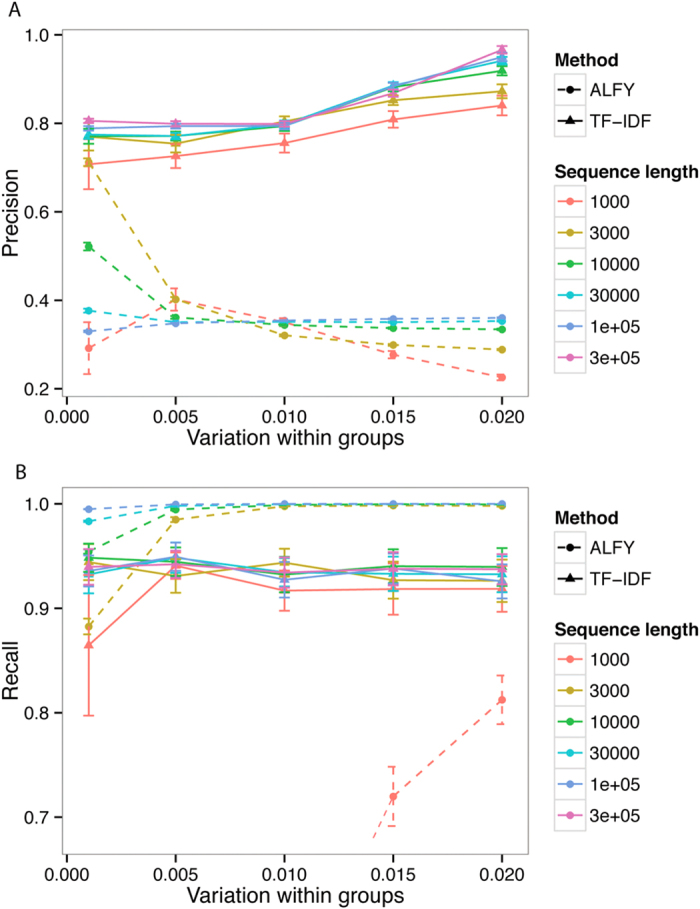
Performance of TF-IDF with variation within groups. Precision (**A**) increases with variation within groups, while recall (**B**) is essentially unchanged. Variation between groups is 0.1, variation post-LGT is zero, and deletion is zero. Error bars are 2× standard error.

**Figure 3:**
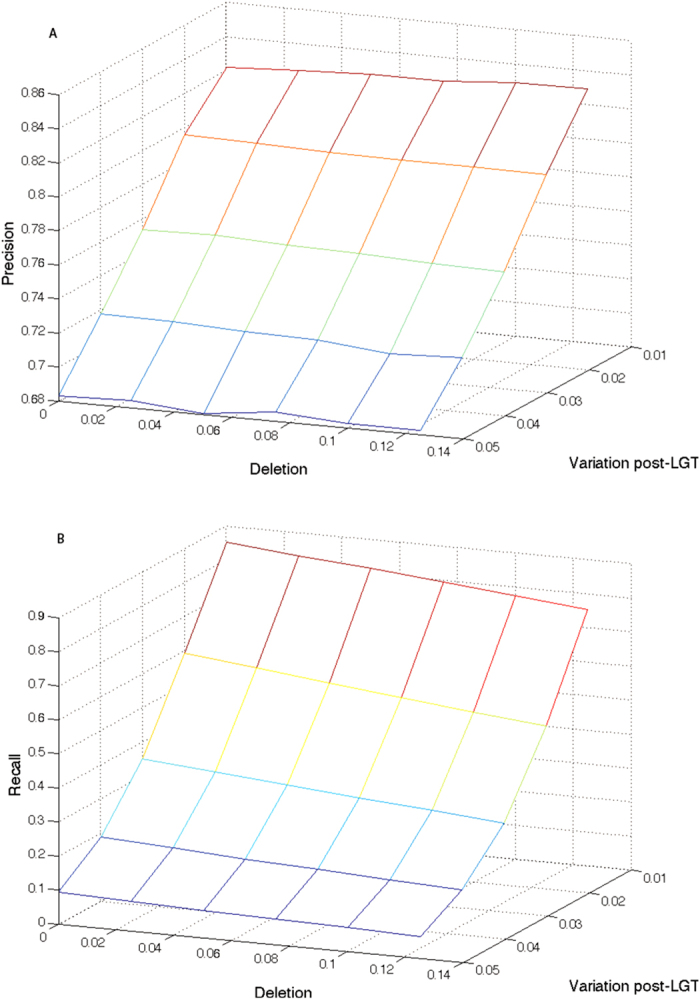
Performance of TF-IDF with variation post-LGT and deletion. Precision (**A**) decreases with variation post-LGT, but is unaffected by deletion. Recall (**B**) decreases greatly with variation post-LGT and slightly with deletion. Variation between groups is 0.1, and variation within groups is 0.01. Sequence length is 300,000 nt.

**Figure 4:**
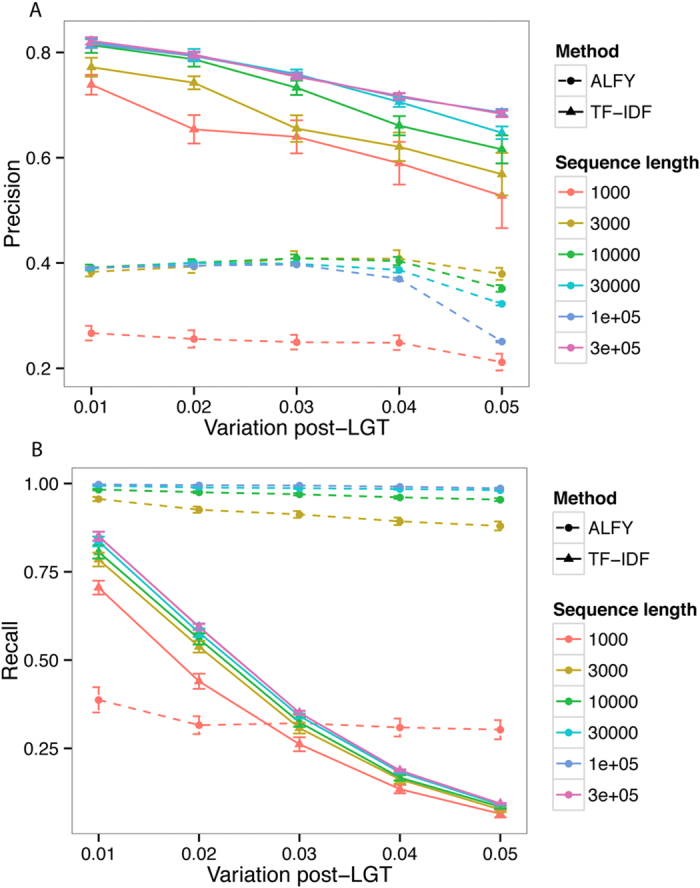
Performance of ALFY with variation post-LGT. Precision (**A**) and recall (**B**) decrease with variation post-LGT. Variation between groups is 0.1, variation within groups is 0.01, and deletion is zero. Error bars are 2× standard error.

As described in Methods, we varied branch length at three stages of the simulation process (variation between groups, variation within groups, and variation post-LGT) and examined the effect on precision and recall. The results are shown in Figs 1, 2, 3, 4 for simulations under the HYK85^26^ model of sequence change; the corresponding plots for F84^27^ are in the Supplementary file. Since TF-IDF does not detect LGT between sequences within a group, for the comparison we ignore such regions that are detected by ALFY; and if an atypical region is equally predicted in several sequences of potential donor groups, we treat this result as a single prediction for the calculation of precision and recall.

Figure 1 shows that when variation between groups is less than 0.05, the average distance accumulated between groups is less than 15%; at this degree of between-group similarity, the precision of our TF-IDF method is low (less than 50%) because the high similarity makes lateral regions harder to distinguish in the recipient group. Precision increases to a high level when variation between groups is above 0.1. Recall is high throughout (approximately 90%) and is less affected by variation; however, at the shortest sequence length examined here (1000), some simulated LGT segments are less than 50 nt in length, too short to contain enough information to make them distinct. As a consequence, recall is significantly lower for this sequence length only.

The precision of ALFY is low, around 0.35, and stable across all branch lengths, but its recall is high. There are two reasons for this. Firstly, ALFY cannot infer the direction of transfer, and may correctly predict one transfer from donor to recipient, but then (erroneously) predict it again from recipient to donor, effectively halving its precision. In the accompanying article^28^ we compare TF-IDF with another directional LGT inference approach^29^ applied to genome-scale empirical data. Secondly, ALFY predicts all most-similar regions as lateral transfers without using a threshold to determine if the similarity is significant or not. As such, it is apparent that ALFY is a useful tool for determining areas which should be further studied for transferred segments, but as a stand-alone detector of LGT it is inferior to TF-IDF. For sequences of length 1000 nt, ALFY’s default sliding window size is too large, leading to reduced performance.

Figure 2 shows the effect of variation within groups on precision and recall. Here, the precision of TF-IDF increases as variation increases. As above, the sequences must be sufficiently dissimilar for the TF statistic to support a decision of LGT. Recall is high, and stable when the sequence length is ≥3000 nt. Again, at sequence length 1000, some short LGT events (<50 nt) are ignored, resulting in decreased recall. The precision of ALFY is stable for variation above 0.005, but again low. TF-IDF shows greater stability and better performance than ALFY in almost all cases, and increasingly outperforms it as the variation increases. As in Fig. 1, ALFY displays better recall than TF-IDF at sequence lengths greater than 1000 nt, but the gap is not large. When the variation within groups is low and the sequence length is short (1000 nt), ALFY again fails to detect most LGT events, leading to extremely low recall (see Supplementary file).

Figure 3 shows the performance of TF-IDF against variation post-LGT and deletion rate for sequences of length 300,000 nt. Plots for other sequence lengths are similar in nature and can be found in the Supplementary file. As variation increases, both precision and (especially) recall decrease substantially, as substitutions progressively obscure the regions of lateral origin. When the branch length post-LGT reaches 0.05 (*i.e.* one nucleotide in ten is expected to have changed, as this is a two-level tree), almost all *k*-mers (for *k* = 40) have been changed, whether in lateral regions or not. In this case, all *k*-mer based methods, including TF-IDF, will fail (and indeed, even alignment-based methods will struggle).

As the amount of deletion increases, precision remains stable and recall decreases slightly. Deletion can move an LGT segment within a sequence, or delete part (or parts) of it. Moving an LGT region does not change its *k*-mers, so this will not affect the performance of TF-IDF. Deletions within a lateral region affect only the immediately adjacent *k*-mers, with little effect on precision unless the region becomes so fragmented that *k*-mer counts are reduced to the point where they are ignored by TF-IDF, degrading the recall.

**Figure 5:**
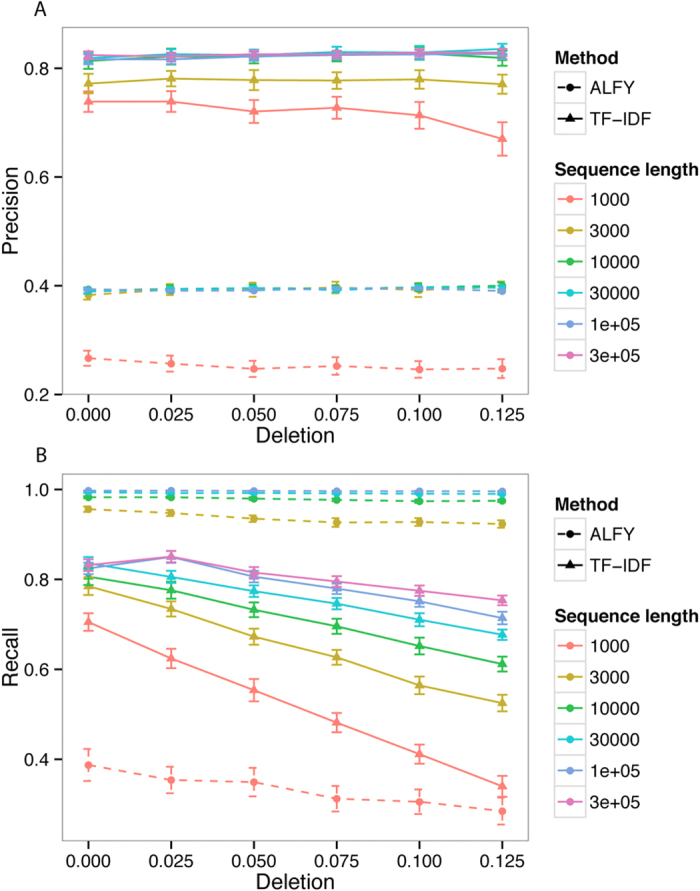
Performance of ALFY with deletion. Precision (**A**) is stable with deletion. Recall (**B**) decreases with deletion. Variation between groups is 0.1, variation within groups is 0.01, and variation post-LGT is 0.01. Error bars are 2× standard error.

Precision and recall increase slightly with sequence length, but length does not appear to interact substantially with the substitution-rate parameters. Since there is no interaction between variation post-LGT and deletion (Fig. 3), we can fix one of these parameters and vary the other. Figure 4 shows that for TF-IDF and ALFY, both precision and recall decrease as variation post-LGT increases. The precision of ALFY is worse than that of TF-IDF, but its recall is higher and more stable. When deletion is varied (Fig. 5), precision is stable except at sequence length 1000, while recall decreases slightly for TF-IDF. As before, TF-IDF is more precise than ALFY, whereas ALFY exhibits higher recall (except at sequence length 1000).

### *k*-mer size

**Figure 6:**
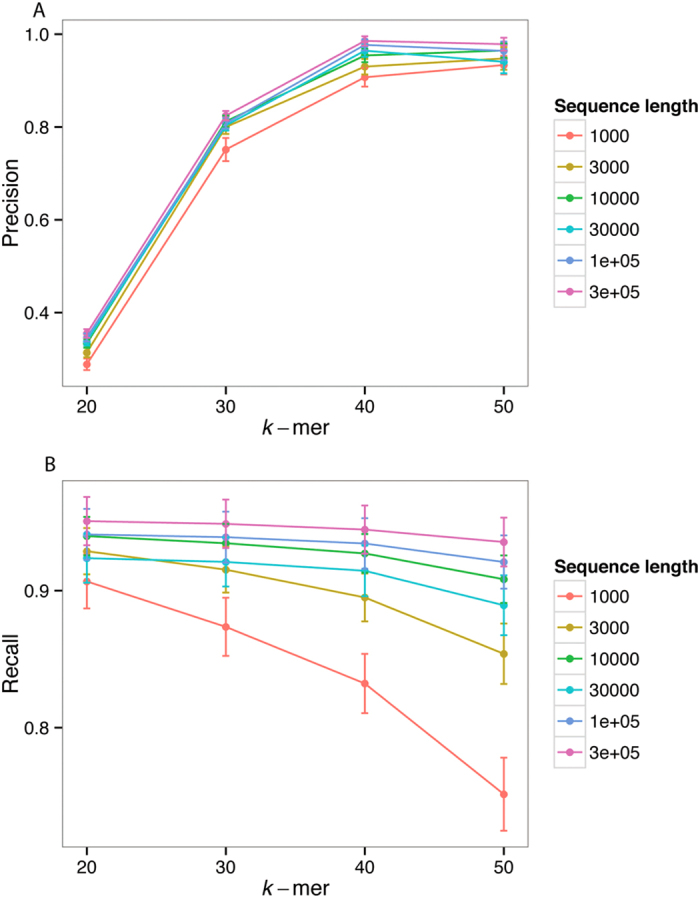
Performance of TF-IDF with *k*-mer size. Precision (**A**) increases with *k*, while recall (**B**) decreases with *k*. Error bars are 2× standard error.

*k*-mer size also affects the performance of TF-IDF. As shown in Fig. 6, precision increases with *k*, but recall decreases. This effect is roughly consistent for every sequence length we examined. The two plots indicate that in this simulation, precision and recall are best balanced at *k* = 40. Indeed, in our experience (as shown and unpublished) *k* = 40 is a useful default setting, in the absence of conditions that argue otherwise. However, if LGT is sufficiently obscured by substitution such that nearly all *k*-mers are unique, TF-IDF will not be able to find sets of *k*-mers that appear frequently in distant groups, and no LGT will be predicted. In such cases, shorter *k* may give better performance. Note that larger *k* imposes a greater memory cost, and more computational time is spent indexing unique *k*-mers.

### Computation time

**Figure 7:**
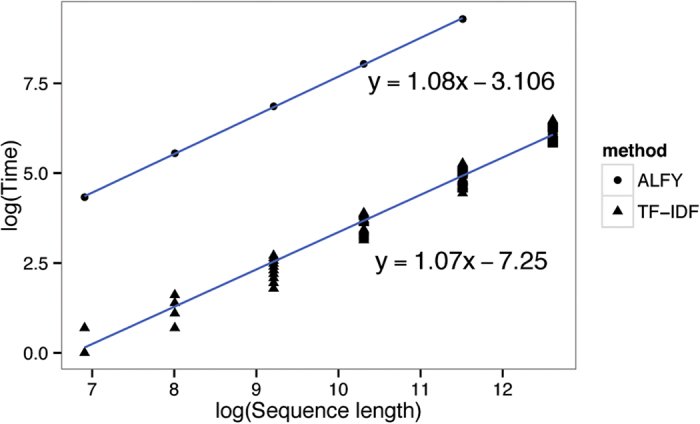
Log-log plot of sequence length against walltime. See text for details.

Figure 7 compares computation time (walltime) for various sequence lengths *L* for ALFY and TF-IDF. All experiments were done on a virtual machine with a single AMD Opteron 2.3-GHz processor and 256 GB memory. As noted below, TF-IDF is expected to scale as *O*(*nL log U*), where *U* is the number of unique *k*-mers in the dataset. *U* is highly dependent on variation at all levels of the simulation, which also leads to variation of time consumption in each experiment; if the final sequences are sufficiently dissimilar, we expect *U* to increase as the number of possible *k*-mers in the dataset, *i.e.* as *nL*. Thus, we expect the time to have an *O*(*L log L*) dependence on *L*, and this is verified in Fig. 7; the slope of the fitted line is 1.07. For ALFY, the time consumption is *O*(*n*^2^*L*) for detecting LGTs between all sequences in a dataset. In a dataset with tens of sequences or more, ALFY will take much longer than TF-IDF, and this is shown in Fig. 7.

**Figure 8:**
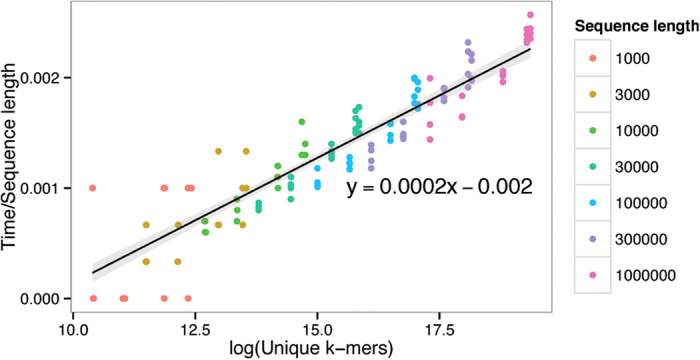
*Log U* against time divided by sequence length. The slope of the regression line is 0.0002, and the grey area is the 95% confidence interval.

Figure 8 shows how walltime depends on *U*. As above, we expect time divided by *L* to have a linear relationship with *log U*, and this is clearly shown.

### Analysis of an empirical dataset

We also tested our algorithm on an empirical dataset that had previously been examined by the developers of ALFY^15^. We used a subset of their dataset, seven genomes of *Staphylococcus aureus*, because this dataset contains strong group information (six genomes from Clonal Complex 8 (CC8) and one multi-drug resistant strain from CC30, *S. aureus* MRSA252) and showed LGT in their analysis. We investigate potential LGT into *S. aureus* TW20, a member of CC8, from MRSA252.

Setting *k* = 40, we identify 1421 regions of TW20 as of lateral origin. Many of these are short and, in this simple example (where the donor group is of size 1, reducing the efficacy of the IDF component) potentially due to noise; but 173 are of length ≥2000, 52 of ≥4000 and 20 of ≥6000 nt (Table 1). It is unclear how to optimise selection of the length threshold, but setting it at ≥2000 nt we infer as lateral 35.6% of the genome, which incorporates 67% (4/6) of the TW20 penicillin-binding genes, and ≥50% (*i.e.* >1.5-fold over-representation) of the annotated genes encoding efflux proteins (2/4), metalloproteinases and -peptidases (3/3), permeases (31/45) and uptake proteins (2/4), types of functions known to be mobilised by LGT^11^,^30^. For details see Supplementary Table S1. By contrast, hypothetical proteins, which might be expected to show no bias for or against lateral origin, are not enriched at any of the length thresholds mentioned above. Ribosomal proteins, which are not expected to be lateral (Jain *et al*.^30^), are rarely represented in our lateral regions (8/60). Phage proteins are not represented in our detected lateral regions; recalling that our approach can discover LGT only *within* the dataset, these results might accurately reflect the history of genetic relationships among these seven genomes. Scope remains for further analysis with other empirical data, and with different settings for *k* and gap size.

**Figure 9:**
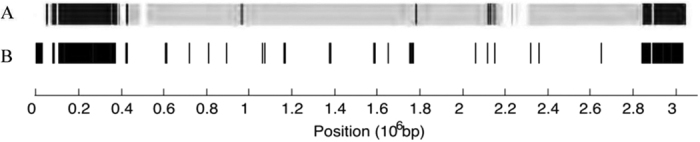
Comparison of TF-IDF and ALFY with an empirical dataset. Both A and B represent the genome of *Staphylococcus aureus* TW20. A shows the result of ALFY analysis^15^; regions inferred to have been transferred from MRSA252 are represented in black, while regions homologous between TW20 and USA300.TCH15156 are shown in grey. B shows the result of TF-IDF analysis. TF-IDF can infer LGT only from outside the target group, so no region is in grey. Both plots were generated from analysis of the seven *S. aureus* genome dataset.

Both our TF-IDF method and ALFY identify most of the genomic region from 2.80–0.42 Mb (TF-IDF) or 2.8–0.5 Mb (ALFY) as lateral (Fig. 9); this region includes two transposons, SCC elements and genes encoding methicillin and penicillin resistance. Robinson and Enright^31^ hypothesised that the methicillin resistance, at least, had been transferred from CC30 into a CC8 background as part of a large chromosomal replacement. The region from 1.75–1.80 Mb includes the transposon Tn*554*^25^, which encodes resistance to erythromycin and spectinomycin. A region from 2.11–2.15 Mb incorporating a number of annotated phage genes was likewise identified. Regions identified as lateral by TF-IDF but not by ALFY include 1.06–1.17 Mb (transport protein genes) and 2.64–2.65 Mb (a transporter and a member of the TetR family of regulatory proteins, which control the expression of genes involved in multidrug resistance and pathogenicity).

## Discussion and Conclusion

We have developed a fast alignment-free method to infer LGT events. Our method is based on TF-IDF, one of the most important methods used in information retrieval. TF-IDF has been widely applied in search engines, document classification and related applications including relevance decision-making. Here we apply TF-IDF to sequence analysis for the first time, treating a sequence or genome as an article and each *k*-mer as a word. Using simulated datasets, we show that TF-IDF can effectively find LGT events with good precision and recall, outperforming ALFY in most biologically realistic situations. We also analyse an empirical dataset and show that TF-IDF finds essentially all regions identified by ALFY as of lateral origin. TF-IDF further detects other regions that, based on annotated gene content, may also have arisen *via* LGT. Our method is alignment-free and scales very well in both length and number of sequences, *i.e.* to many entire genomes. It is worth noting that in each simulated dataset, all sequences share the same length and group size. For the empirical dataset, the group sizes and lengths of the seven *S. aureus* are of the same magnitude. For this reason, we did not normalise the count of *k*-mers in the IDF step. However, in other empirical datasets the sequence length and group size may vary greatly, and normalisation might be considered^28^.

Our method is purely data-driven, its performance relying strongly on sequence and group information in the dataset. In our simulations, when sequences are relatively similar within-group (variation 0.005–0.02) and relatively dissimilar between-group (variation >0.1), group boundaries are clear, and the precision and recall of our algorithm is high. When speciation is modest (<0.05), within-group divergence high (<0.1) or LGT events obscured by subsequent evolution (>0.02), TF-IDF loses precision in inferring LGT events.

In the accompanying article^28^ we apply this method to larger empirical datasets. TF-IDF could further be applied to environmental data, e.g. to study the flow of genetic material in communities and across the biosphere. We anticipate that significant scope remains for further algorithmic and implementational improvements.

## Methods

### Notation

Here we establish some notation. We start with a dataset of *n* sequences, each of length *L*. For empirical datasets (and for some approaches to simulation) the length may vary among sequences; in those cases we use *L* to denote the average length. The sequences in the dataset are divided into *m* groups corresponding to closely related genomes (*e.g.* belonging to the same clonal group, species or genus). We denote each sequence as *S*_*i,j*_, where *i* = 1, 2, …, *n* is the number of the sequence in the dataset and *j* = 1, 2, …, *m* is the number of the group to which the sequence belongs. The number of sequences in group *j* is denoted by *h*_*j*_.

Our method proceeds by comparing substrings (words) of a fixed length *k*, called *k*-mers. We encode each sequence as a frequency vector of *k*-mers, counting only those *k*-mers that actually appear in the sequence, and denoting the number of unique *k*-mers appearing in the dataset by *U*. In general, *U* is much smaller than 4^*k*^, the total number of all possible *k*-mers.

Although we illustrate our approach here using nucleotide sequences, the method is easily adapted for amino acids, requiring only a change of alphabet.

### TF-IDF on texts

As mentioned above, TF-IDF was introduced to indicate the topic of a document, and distinguish that document from others in the same corpus for a specific query. The classical usage of TF-IDF is as a smart retrieval system and for automatic document categorisation^32^,^33^,^34^. A variant uses prototype vectors to calculate relevance between documents with a nearest-neighbor learning method^35^. PrTFIDF^36^ is an improved version of TF-IDF founded on a probabilistic model for text categorization, and there are other variants for calculating TF-IDF^37^. In recent years, TF-IDF has also been applied in other areas including decision-making and sentiment analysis^24^,^38^.

TF-IDF is widely used in text mining and information retrieval because it allows the identification of terms that are characteristic of (and hence important for) one text or a set of texts. It is not sufficient for a term to be frequent in a text (TF); it must also be rare in other texts in the corpus (IDF). Importantly, IDF depends only on the occurrence of terms, not on their numerical frequencies. Drawing on analysis of documents in three independent domains, Salton and Yang^39^ identified five situations relevant to the performance of TF-IDF:

1. Terms that appear frequently across a corpus contribute little to performance because they do not discriminate between relevant and non-relevant documents;
2. Terms that appear in a moderate number of texts and show somewhat skewed distributions provide good retrieval performance;
3. Terms with sharply skewed distribution occurring in very few documents are important only for those documents;
4. Rare terms are important for the few queries and documents in which they occur; and
5. Terms of low or moderate frequency, but with a flat distribution across documents, are similarly useful for the documents in which they occur.

Classically, the frequencies of terms in a corpus follow a power law (Zipf law), in which case TF-IDF performs well. However, TF-IDF can perform adequately even when this is not the case: TF-IDF requires only that terms relevant to the query are distributed intensively in a subset of documents within the corpus^23^,^40^; this might include the query terms themselves (*e.g. happy*), or related terms in the corpus (*pleased*, *delighted*).

### TF-IDF on sequences

Molecular sequences have long been analogised with natural language^41^ or treated as texts^42^. Alternatively, both molecular sequences and texts have been subsumed within a broader class of objects^43^. The analogy is not precise: in sequences, “terms” must be recognized computationally, *e.g.* by extracting *k*-mers. Fast approaches exist for extracting *k*-mers^44^,^45^, and *k*-mer distribution in empirical sequences has been studied at some length^46^,^47^,^48^. Like words in text, short *k*-mers (*k* between three and eight) in DNA sequences show Zipf-like scaling^49^, although this is not sufficient to confirm DNA sequences as a natural language^50^.

Although there is dispute whether DNA is a language or not, some methods in text mining have been successfully applied to DNA analysis. For example, the first (to our knowledge) software to identify lateral transfer in biological datasets^51^ was repurposed from the analysis of textual contamination in manuscripts, which in turn was built on software for phylogenetic inference from DNA sequences^52^ (PHYLIP).

Sequences (genomes, genes, proteins) do, however, differ from texts in some properties. For example, *k*-mer frequency distributions in sequences are usually much flatter than term frequencies in texts. Experience from text mining indicates that this is not critical, but this remains to be explored and is in fact a goal of the current work. In the specific application here, genomic regions of lateral origin are expected to have *k*-mers that appear frequently in genomes of the donor taxon, but rarely in the host. This is analogous to conditions 2 and/or 4 above^39^.

Our algorithm works by comparing the frequencies of identified *k*-mers in a group of sequences (our TF) with their frequencies in other groups (our IDF). If a *k*-mer in one sequence is prevalent in a different group but not in its own, then it may have arisen by LGT from the group in which it is prevalent, and the direction of the transfer should be from that (donor) group to the recipient sequence. We compare these TF and IDF statistics to appropriate thresholds to optimize detection performance, *i.e.* to balance precision and recall.

Our algorithm consists of four steps: extracting all *k*-mers from genomes within one dataset calculating inverse document frequencies, constructing potential LGT segments, and calculating term frequencies. For pseudocode, see the Supplementary file.

### Extracting *k*-mers

To extract *k*-mers we scanned all the genomes, incrementing one nucleotide at one time. If the genome length is *L*, then *L*-*k* + 1 *k*-mers are found. Unique *k*-mers were indexed in a red-black tree^53^ for further searching.

### Calculating IDF

To calculate the inverse document frequency, we construct an *n* × *m* relationship matrix *R*, denoting the frequency (number of occurrences) at which *k*-mers in each sequence appear in each group. Each row in *R* corresponds to a sequence, and each column corresponds to a group. Suppose sequence *i* consists of *k*-mers *w*_*i*,1_, *w*_*i*,2_, …, *w*_*i,L-k+1*_. If the word *w* appears in group *j* with frequency *f*_*j*_*(w)*, then the entries of the relationship matrix are


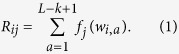


The entries in *R* are our IDF values. The larger the *R*_*ij*_, the more likely that sequence *i* contains a region transferred laterally from group *j*. Note that this is in contrast to the original definition of IDF, where a higher IDF indicates that the word appears less frequently in other documents.

To detect potential lateral-transfer events, we compare the IDF values against a threshold *t*. This threshold is the average value of all entries in *R*:


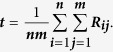


IDF values that are above the average are used for further analysis.

### Constructing potential LGT segments

We then mark potential lateral segments in each sequence. For each sequence *i* and group *j* with a sufficiently high IDF value, we examine each *k*-mer in sequence *i* to see if it appears in group *j*. Then we join all consecutive *k*-mers which do, forming potential lateral segments. Because mutations or other genomic events may disrupt the perfect matching, we allow gaps between blocks of *k*-mers of size up to a threshold *G*. Here we set *G* = 2*k*, a value at which the total number of detections is not greatly affected in real application^28^. We then assess the significance of these potential lateral segments using term frequency.

### Calculating TF

For each potential lateral segment *σ* in a sequence, we calculate the frequency (number of occurrences) at which each of its component *k*-mers appears in sequences of its own group, say *j*. Our TF statistic for *σ* is the sum of these:


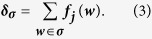


If *δ*_*σ*_ is higher than some threshold, then *σ* occurs frequently in its own group, and as such is considered not to be the consequence of a lateral event; otherwise it is considered to be of lateral origin.

To set the threshold, we calculate the average frequency of all unique *k*-mers in the recipient group *j*, denoted by *τ*_*j*_. Then we compare *δ*_*σ*_ to *lτ*_*j*_, where *l* is the number of *k*-mers contained in the segment. If *δ*_*σ*_ is smaller, we consider *σ* to have been transferred laterally from the other group into this sequence. Other approaches to setting the threshold are possible, but we do not consider them here.

Note that our method considers lateral transfers only within the dataset; like most other LGT methods, it is silent on potential transfers from sources external to the dataset. In addition, it can detect transfers only between groups, not between sequences in the same group.

### Runtime analysis

The computational complexity of the algorithm is dominated by extraction of the unique *k*-mers in the dataset. To find these, we scan each of the *n* sequences of length *L*. As each unique *k*-mer is found it is added to a library, which is stored in a red-black tree^53^. A red-black tree is an approximately balanced tree, which guarantees that searching and insertion are efficient. On average, this step takes *O*(*nL log U*) time, where *U* is the number of unique *k*-mers stored in the tree. The frequency of each *k*-mer is also computed at this time. The remaining calculations are much quicker because most of the frequency (*f)* terms are zero. Thus for biological sequences of standard complexity, runtime increases about log-linearly with sequence length. Note that the *k*-mer profiles of each sequence could in principle be stored and retrieved for future use.

### Implementation

We have implemented this algorithm in C++. The program can be compiled using GCC 4.8.2 and run on Unix, Unix-like and Windows platforms. We use the *map* template from STL (Standard Template Library) to index all distinct *k*-mers in a dataset. The inner implementation of *map* is a red-black tree^53^.

### Comparisons with ALFY

ALFY finds putative homology (shared DNA segments) between pairs of sequences by matching shustrings (shortest unique substrings). If a match is found with a region in an otherwise distant sequence, it will be judged as a potential lateral transfer. This method shows high efficiency and effectiveness for LGT detection^15^,^16^, so we use it to benchmark our method.

The inputs to both TF-IDF and ALFY are sequences. For TF-IDF the group information is compulsory, while ALFY requires a query sequence and subject sequences. Both TF-IDF and ALFY can process DNA sequences; TF-IDF can also process amino-acid sequences, but ALFY does not currently implement evolutionary models of amino-acid change. Only *k*-mer frequencies will be taken into consideration for calculating the value of TF-IDF.

In TF-IDF, if a *k*-mer has low a frequency in its own group but high frequencies in other groups, then this *k*-mer will be judged atypical. A set of contiguous atypical *k*-mers will be inferred as lateral, with the direction of the transfer from the *k*-mer prevalent group. In contrast, ALFY computes the average shustring length between segments of only two sequences at a time. The longer the average shustring, the closer the two segments; and if the sequences themselves are otherwise distant in the reference tree, the segment in question will be inferred as lateral, without any implication of which sequence was donor or recipient.

If the sequences are grouped such that each group is compact and boundaries between groups are clear, then TF-IDF should find lateral segments easily. ALFY does not use group information, so grouping does not affect its performance.

The computational complexity of TF-IDF is *O*(*nL log nL*), where *n* is total number of sequences in a dataset, and *L* the average length of sequences in a dataset. The computational complexity of the ALFY algorithm is *O*(*nL*). However, TF-IDF will process all sequences and infer all potential lateral regions over an entire dataset, whereas ALFY makes all pairwise comparisons between a single query sequence and the others. For fairness of comparison, all sequences in a dataset should be set as queries to find all LGTs in a dataset, in which case the complexity of ALFY increases to *O*(*n*^2^*L*), which in practice is much slower than TF-IDF.

### Simulation of datasets

In order to test the performance of TF-IDF in different situations, and to compare with ALFY, we simulated datasets under the HYK85^26^ and F84^27^ evolutionary models. Our simulation process is as follows:

1. We start with one random sequence, which will become the ancestor of all sequences in the dataset. We vary the length *L* of this sequence from 1000 to 300000 characters to simulate sequences from a single gene to a significant part of a genome (but our algorithm can be applied to sequences of any length).
2. To establish phyletic groups (*i.e.* to simulate speciation), the ancestral sequence is allowed to evolve along a balanced binary tree with four levels of equal branch lengths, using the evolutionary model. The branch length varies from 0.01 to 0.20 (substitutions per site) in steps of 0.05. We refer to this parameter as *variation between groups*.
3. To populate these groups with sequences, each descendant (leaf) in the initial tree (above) is allowed to evolve along another phylogenetic tree under the same evolution model. Again we use a balanced binary tree with four levels of equal branch length, which vary from 0.001 to 0.020 in steps of 0.005. We refer to this parameter as *variation within groups*.
4. We then simulate LGT events between groups. For the sake of simplicity, here we make transfers only into sequences in Group 1. We fix the number of LGT events at 20, with lengths normally distributed around mean 0.1 of the sequence length, and standard deviation half that amount. For each simulated event the recipient sequence (in Group 1) is selected at random, with (typically) several sequences receiving multiple transfers and others receiving none. Transfer events overwrite the equivalent positions in the recipient sequence, but (to simplify our simulation) cannot themselves be subsequently overwritten. Five of the 20 LGT events are simulated to come from the group (of 16 sequences) arising from the most-recent common ancestor on the binary tree (from Step 2), five from descendants of the second-most recent ancestor (32 sequences), five from the third (64 sequences) and five from the deepest bifurcation (128 sequences). Thus the probability of transfer decreases with increasing distance (on the tree) between donor and target.
5. In a final evolutionary process, we further evolve each of the 256 sequences along a balanced two-level tree, with branch lengths varying from 0 to 0.1 in steps of 0.025. We refer to this parameter as *variation post-LGT.*
6. In some simulations, Step 5 also includes a stochastic process (implemented by using a shell script to call ALF^54^, not to be confused with ALFY) which deletes from 0 to 10% of a sequence. The proportion was varied using the *deletion rate* setting in ALF, while keeping *deletion length distribution* at its default value. We refer to this parameter as *deletion*. We did not simulate duplications here because bacterial genomes contain very few repetitive components.

After the above steps, we select one descendant of each tree to yield our final dataset (256 sequences per simulation).

**Figure 10:**
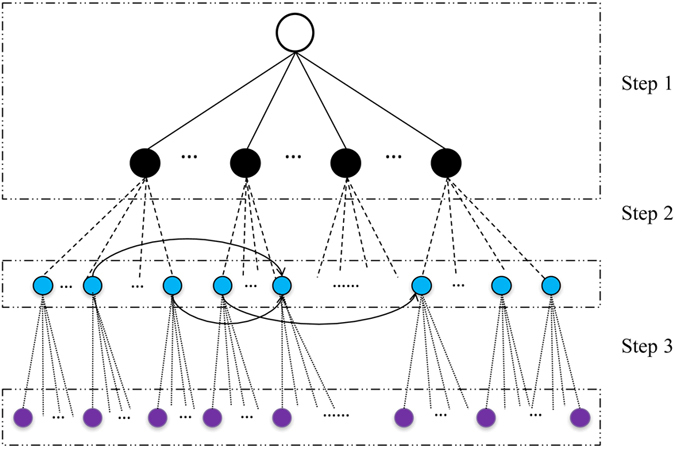
Overview of data simulation. Step 1: The simulation starts with a single ancestor and generates 16 sequences, which serve as ancestors for each group (variation between groups). Step 2: Within each group we generate 16 descendants (variation within groups), then add LGT events between these groups. Step 3: Finally we simulate variation post-LGT, which may include deletion. From each initial ancestor the simulation generates 256 sequences. Symbols: 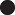 DNA sequences which are ancestors of the sequence groups. 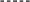 Phylogenetic tree used to generate populations of each group. 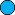 DNA sequences that constitute groups. 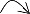 LGTs events are added between them. LGT between two sequences. 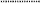 Phylogenetic tree on which the evolutionary process post-LGT is simulated. This process tends to obscure the LGT events. Branch length determines the ‘age’ of the LGT events. Regions of the sequences may be deleted at this step. 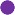 DNA sequences generated by the simulation.

In addition to varying the parameters mentioned above for both TF-IDF and ALFY, for TF-IDF only we also varied the word length *k*, in steps of 10 from 20 to 50. As the number of possible parameter combinations above is very large, at Step 2 we varied only the *variation between groups* parameter while keeping all others fixed at minimal-impact settings. Similarly at Step 3 we varied only the *variation within groups* parameter. For each parameter combination we simulated 50 datasets under the F84 model of sequence change, and 50 under HYK85. This process is depicted in Fig. 10, and is explained in greater detail in the Supplementary file. We also analysed smaller datasets omitting Step 4, to examine whether TF-IDF inferred LGT when none was present; no segments met the IDF (*k*-mers frequent in donor groups) and TF (*k*-mers infrequent in the recipient group) criteria simultaneously, so no LGT was inferred.

### Performance measures

We assessed the performance of our algorithm on simulated data using two measures. Precision is the proportion of inferred LGT events which are real (*i.e.* were actually simulated):


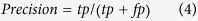


where *tp* and *fp* are the total lengths of all true and false positives respectively. Recall is the proportion of true (simulated) LGTs which were inferred by the algorithm:


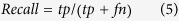


where *fn* is the total length of false negatives (simulated LGTs which were not found).

**Figure 11:**
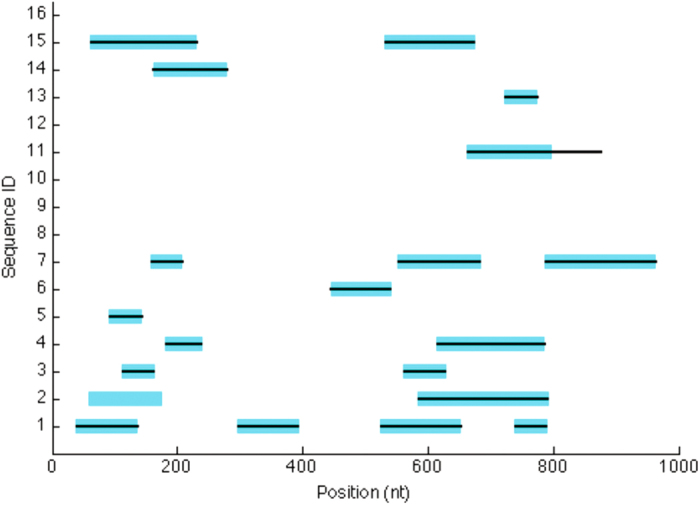
An example of simulated and inferred LGTs. The *x*-axis displays the nucleotide position, and the *y*-axis the sixteen sequences generated in our first (recipient) group. The wide bars show the lateral regions actually simulated, and the narrow black bars the regions inferred as lateral by TF-IDF. Here, variation between groups is 0.1, variation within groups is 0.001, variation post-LGT is 0.01, deletion is zero, *k* = 40 and sequence length is 1000 nt.

Figure 11 illustrates the output of TF-IDF analysis of a simulated dataset, showing the 20 regions of (simulated) lateral origin of which 19 were detected (wholly or in part) by TF-IDF. Positions 797–877 of Sequence 11 represent a false positive inference of LGT, and positions 58–117 of Sequence 2 a false negative. Overall for this dataset (*i.e.* LGT from Groups 2–16 into Group 1), precision was 0.82 and recall 0.95. Complete details (start and end coordinates) are presented in the Supplementary file.

## Additional Information

**How to cite this article**: Cong, Y. *et al*. A novel alignment-free method for detection of lateral genetic transfer based on TF-IDF. *Sci. Rep.* **6**, 30308; doi: 10.1038/srep30308 (2016).

## Supplementary Material

Supplementary Information

## Figures and Tables

**Table 1:** Summary of regions in the *Staphylococcus aureus* TW20 genome (GenBank NC_017331.1) inferred as lateral by TF-IDF.

	2000–3999		4000–5999		6000+		2000+	
	No.	%	No.	%	No.	%	No.	%
LGT regions	121	8.5	32	2.3	20	1.4	173	12.2
Mean size (nt)	2797	—	4782	—	29600	—	6263	—
Median size (nt)	2786	—	4727	—	10496	—	3112	—
Nucleotides	338413	11.1	153009	5.0	592007	19.5	1083429	35.6
Proteins^1^	405	14.6	169	6.1	515	18.5	1071	39.2
Hypothetical proteins	116	14.3	38	4.7	157	19.3	311	38.3

Numbers in the top row refer to the length ranges of segments selected for analysis.

^1^Protein-coding genes fully or partially contained within a region inferred as lateral by TF-IDF.
