# Supplementary material for: A novel alignment-free method for detection of lateral genetic transfer based on TF-IDF
Source: Sci Rep. 2016 Jul 25;6:30308. doi: 10.1038/srep30308 (PMC4958984; doi:10.1038/srep30308)
Supplement: Supplementary Information [file srep30308-s1.pdf]

Supplementary Material

A novel alignment-free method for detection of lateral genetic transfer based on TF-IDF

Yingnan Cong

Yao-ban Chan

Mark A. Ragan

1 Pseudocode for the TF-IDF algorithm

```
Begin
  Recognise all the different k-mers of size k and label as 1, 2, ..., U.
  // Compute Matrix M
  M ← Zeros(n × U)          // Zeros returns an all-zero matrix of the specified dimensions.
  For each sequence i do
    For each k-mer κ in sequence i do
      M(i, L(κ)) ← M(i, L(κ)) + 1          // L(κ) returns the label of k-mer κ.
    End For
  End For
  // Compute Matrix R
  R ← Zeros(n × m)
  For each sequence i do
    For each group j except Θ(i) do          // Θ(i) returns the group of sequence i.
      For each sequence i′ in group j do
        R(i, j) ← R(i, j) + Ω(i, i′)          // Ω(i, i′) returns the number of
                                              // common elements between sequences i and i′.
      End For
    End For
  End For
  t ← avg(R)          // avg(R) returns the average value of elements of R.
  // Compute τ
  τ ← Zeros(m)
  For each group j do
    K ← M(j, °)          // M(j, °) returns the rows that represent the sequences in j
    τ(j) ← numel(K)/numel(unique(K))
  End For
  // Detect LGTs
  (i, j, v) ← Fmax(R)          // Fmax(R) returns the maximum value of R as v, with
                               // corresponding sequence i and group j.
  While v > t
    // Cut sequence i
    ω ← Zeros(m)
    For each k-mer κ in sequence i do
      ω(i) ← ismember(κ,j)          // ismember(κ,j) returns 1 if κ exists in at
                                    // least one sequence of species j, 0 otherwise.
    End For
    TagS ← 0
    TagE ← 0
    Intrpt ← 0
    For each element ζ (p-th) in ω do
      If ζ=1
        If TagS = 0
          TagS ← p
          TagE ← p
        Else
          TagE ← p
        End If
        Intrpt ← 0
      Else
        If Tag ≠ 0
          Intrpt ← Intrpt + 1
          If Intrpt > 2 × k
            Add (TagS, TagE) to f          // f denotes segments of interest
```

$TagS \leftarrow 0$
End If
End If
End If
End For
For each segment ϕ in $\boldsymbol{f}$ **do**
$\varepsilon \leftarrow 0$
For each sequence i' in group $\vartheta(i)$ **do**
For each k-mer κ in ϕ **do**
$\varepsilon \leftarrow \varepsilon + \mathbf{M}(i', L(\kappa))$
End For
End For
If $\varepsilon < \mathbf{\Gamma}(i) \times l$ // *l denotes the length of the fragment, i.e.,* $TagE - TagS$
Add $\boldsymbol{f}$ as a LGT.
// Update Matrix **R**
$\mathbf{R}(i,j) \leftarrow 0$
End If
End For
$(i, j, v) \leftarrow \mathbf{F}\boldsymbol{max}(\mathbf{R})$
End While
End Begin

2 Details of our simulation

The simulation process is as follows:

Step 1: Generate groups

- Generate one random sequence as ancestor of all sequences.
- Set a phylogenetic tree to generate different groups. Here, we use a 4-level full binary tree, thus generating 16 sequences, each of which will become the ancestor of a group. The branch lengths of the tree are identical and control the variation between groups.

Step 2: Generate individuals in each group

- Pick one sequence generated in last step as the ancestor of a group.
- Set a phylogenetic tree to generate individuals. As with the last step, we use a 4-level full binary tree to generate 16 individuals. The branch lengths of the tree are identical and control the variation within groups.
- Repeat the previous two lines until all sequences generated in Step 1 have been used.

256 sequences are generated in this step.

Step 3: Add LGT events

- Set the total number of LGT events. We use 20 in our experiments.
- Determine the distribution of LGT events. We take LGTs only to group 1 from other groups. The LGT donors are distributed evenly (5 each) among the following four sets: group 2, groups 3 and 4, groups 5 to 8, and groups 9 to 16.

Step 4: Evolve post-LGT

- Set a 2-level full binary tree. The branch lengths are identical and control the variation post-LGT.
- Let every sequence evolve following this tree, and add deletion simultaneously to get 4 descendants.
- Randomly pick one descendant from each sequence.

This generates a final simulation dataset with 256 sequences.

Parameters

Variation between = 0.01, 0.05, 0.1, 0.15, 0.2, within = 0.01, post_LGT = 0, deletion = 0, k=40
Variation between = 0.1, within = 0.001, 0.005, 0.01, 0.015, post_LGT = 0, deletion = 0, k=40
Variation between = 0.1, within = 0.01, post_LGT = 0, 0.02, 0.04, 0.06, 0.08, 0.1, deletion = 0, 0.025, 0.05, 0.075, 0.1, 0.125, k = 40
$\pi_T = \pi_c = \pi_A = \pi_G = 0.25, \kappa = 2$ under HYK85 model.
$\pi_T = 0.291, \pi_c = 0.275, \pi_A = 0.304, \pi_G = 0.130, \kappa = 2$ under F84 model.

3 Coordinates of simulated and inferred LGT regions in Group 1 for Figure 2

Simulated LGT		Inferred LGT		Recipient (Sequence)	Donor (Group)
**Start**	**Length**	**Start**	**Length**		
297	95	297	97	1	16
182	56	182	56	4	16
786	177	786	177	7	15
614	170	614	172	4	15
532	142	532	143	15	11
552	131	552	131	7	6
157	50	157	50	7	8
739	50	739	50	1	5
722	51	722	53	13	6
92	50	92	53	5	7
445	95	444	96	6	3
112	50	111	52	3	4
163	115	161	118	14	4
62	167	62	169	15	3
585	206	585	206	2	3
662	134	662	215	11	2
562	66	562	66	3	2
525	127	525	127	1	2
39	96	38	98	1	2
58	117	--	--	2	2

4 Performance of TF-IDF under the F84 model (variation between and within groups)

Here we replicate the TF-IDF analyses shown in Figures 3 and 4, under the F84 evolution model.

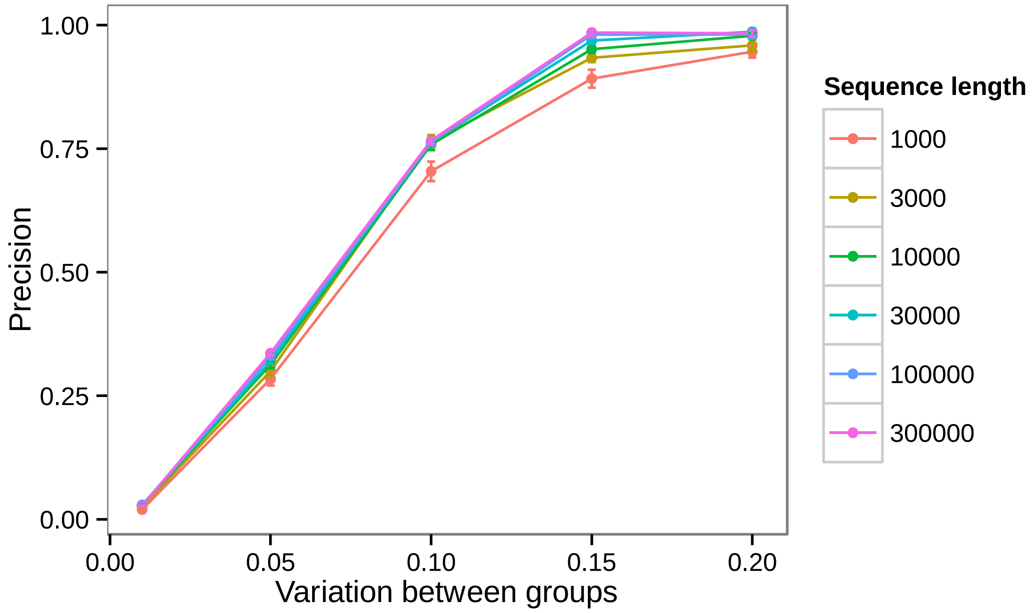

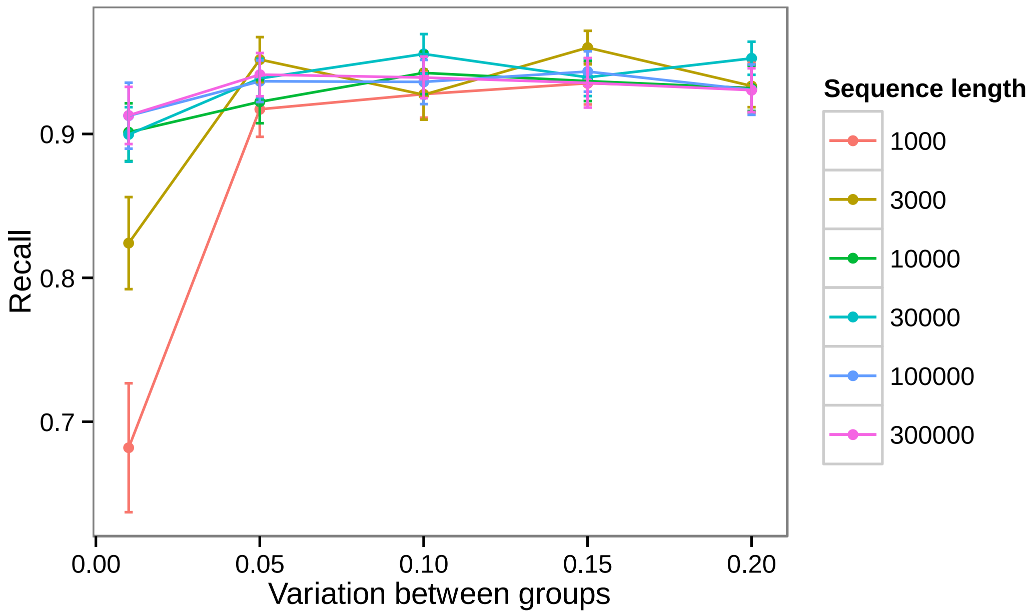

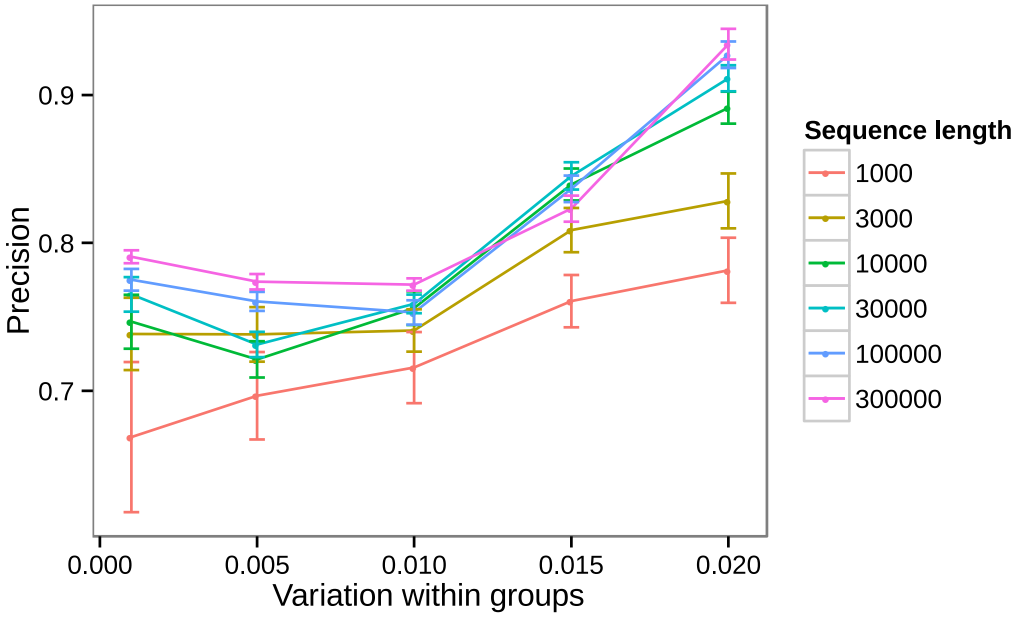
Precision
0.9
0.8
0.7
0.000
0.005
0.010
0.015
0.020
Variation within groups
Sequence length
1000
3000
10000
30000
100000
300000

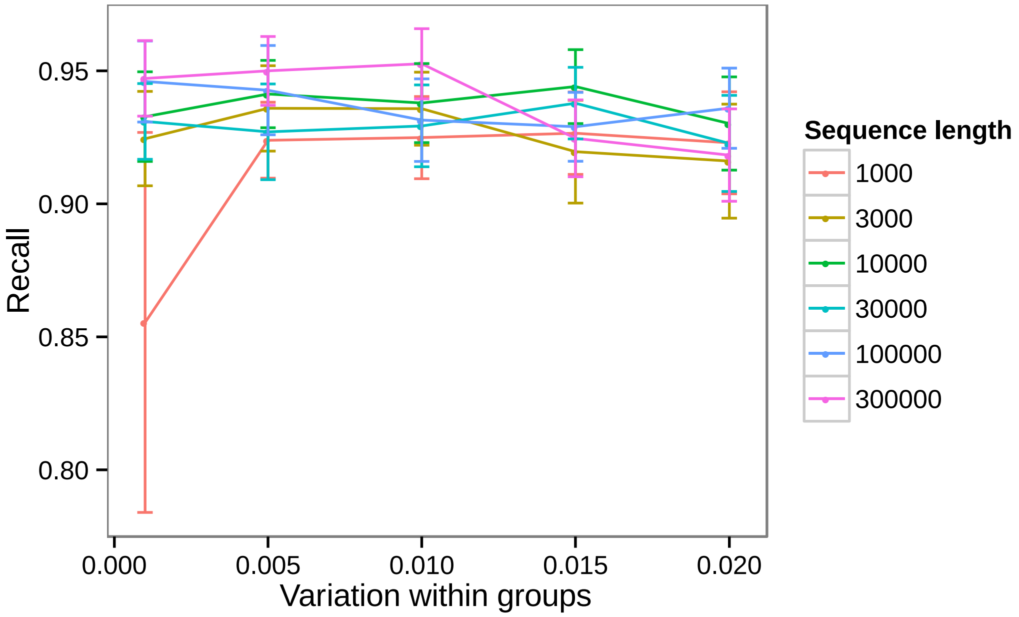
Recall
0.95
0.90
0.85
0.80
0.000
0.005
0.010
0.015
0.020
Variation within groups
Sequence length
1000
3000
10000
30000
100000
300000

5 Full comparison of recall of TF-IDF and ALFY for variation within groups

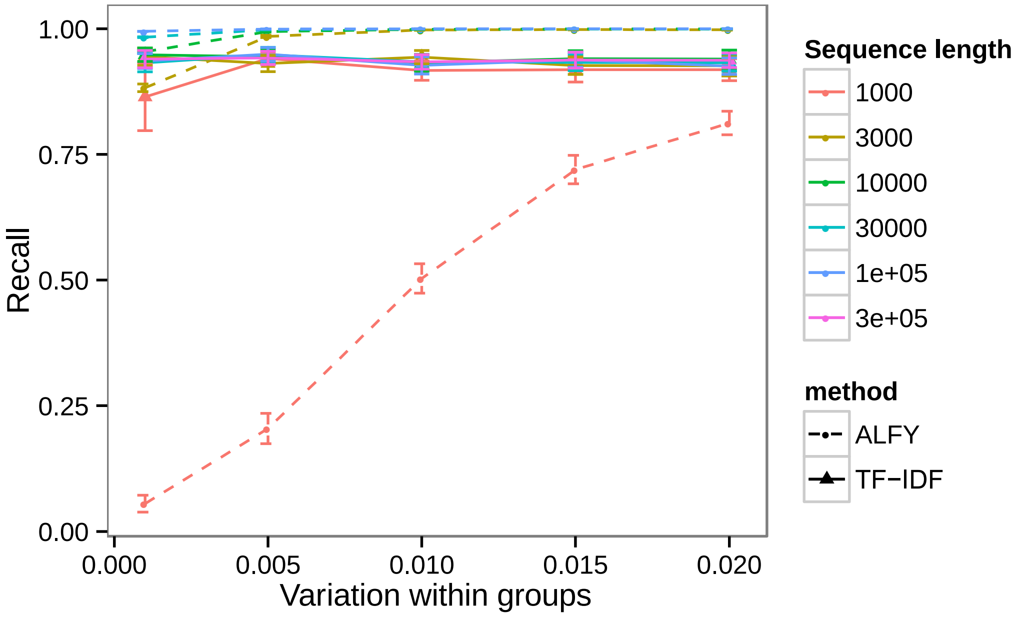

6 Performance of TF-IDF with variation post-LGT and deletion with different sequence lengths.

Performance on variation post-LGT and deletion (sequence length = 100,000 nt)

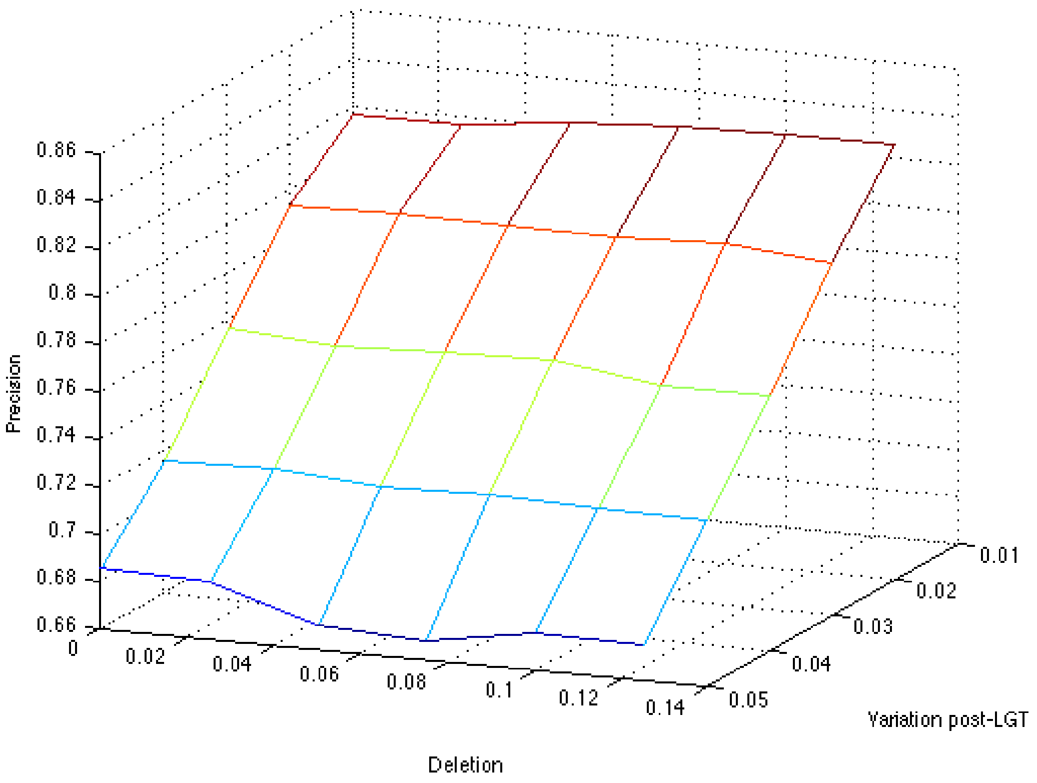

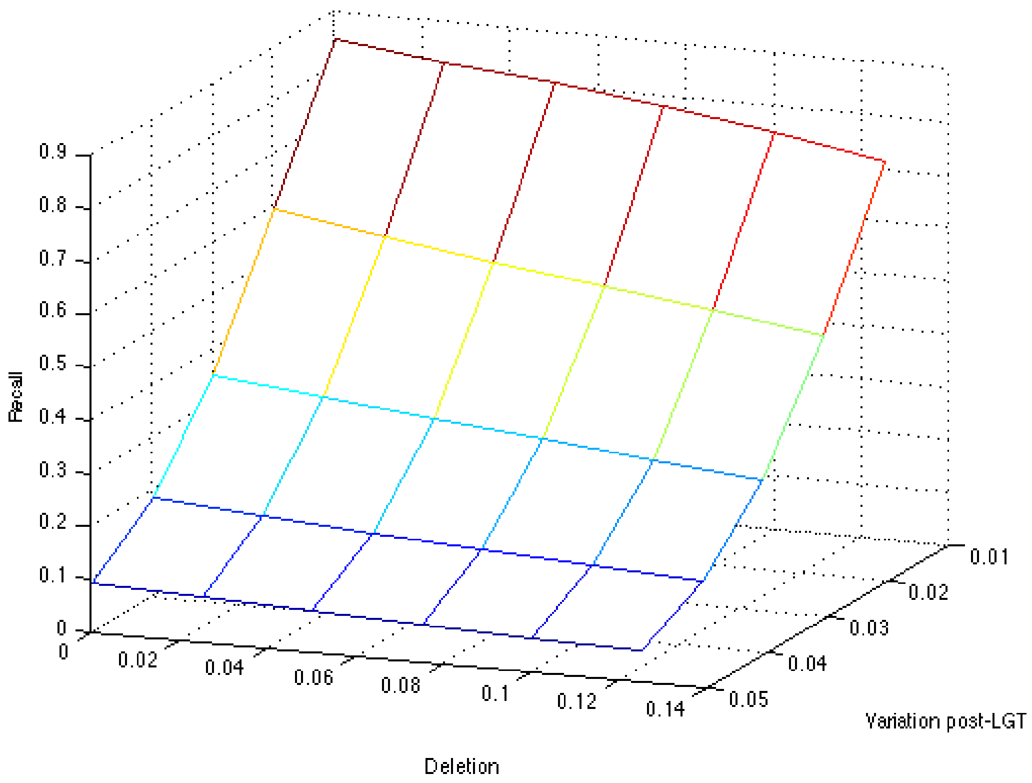

Performance on variation post-LGT and deletion (sequence length = 30,000 nt)

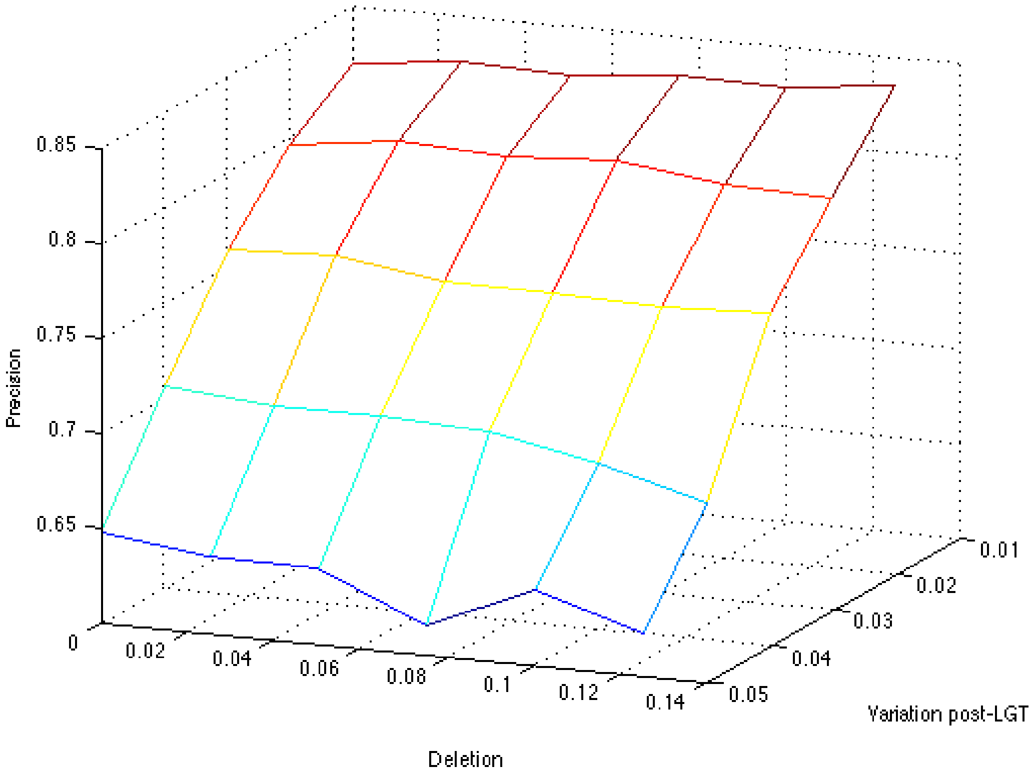

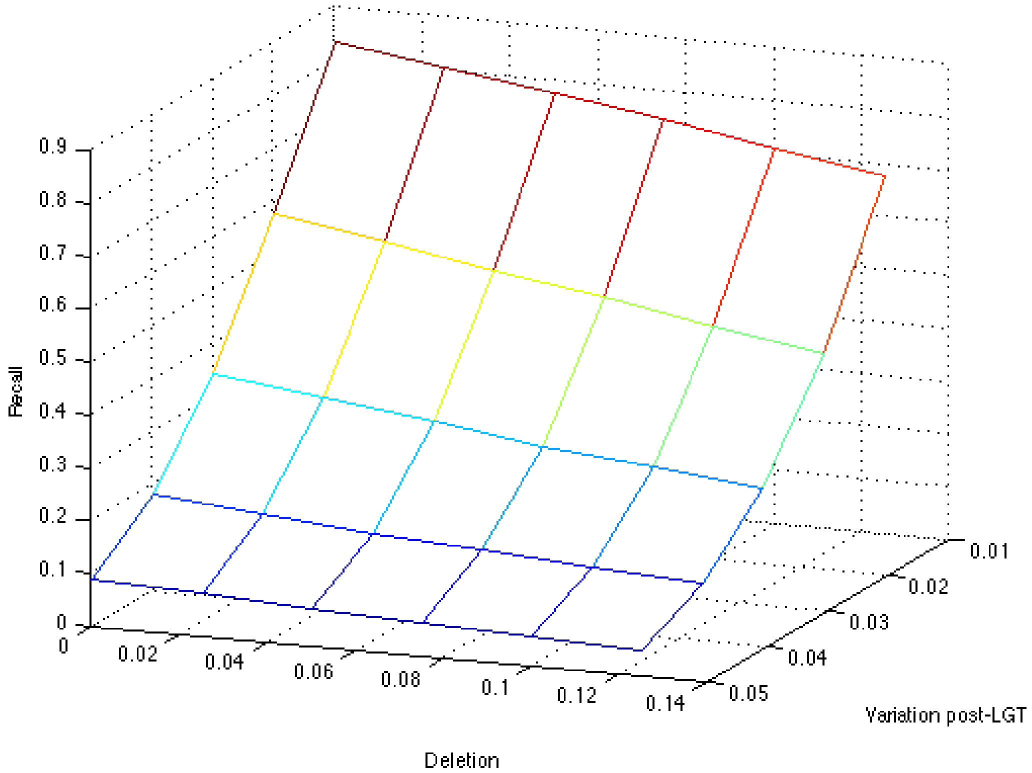

Performance on variation post-LGT and deletion (sequence length = 10,000 nt)

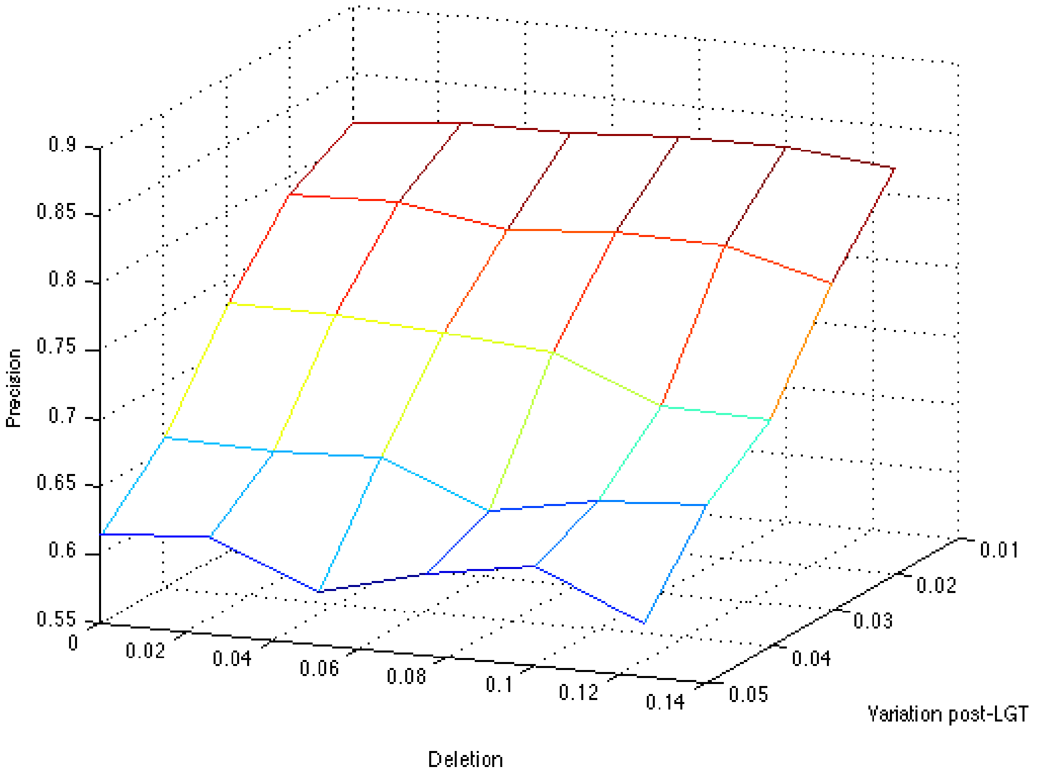

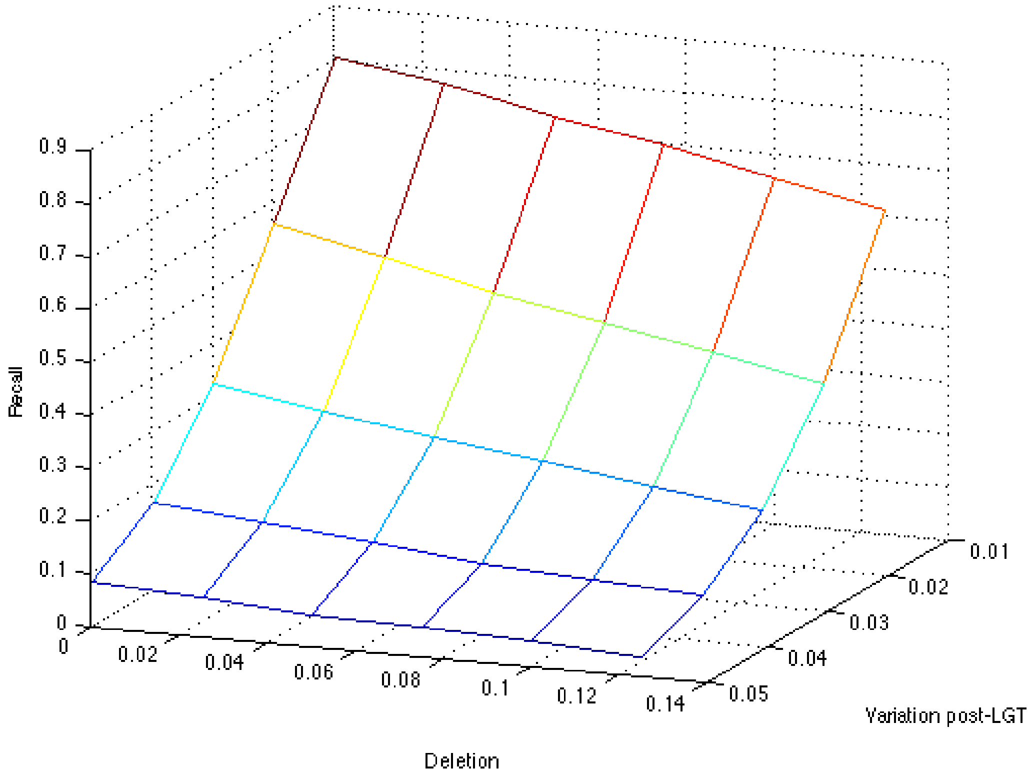

Performance on variation post-LGT and deletion (sequence length = 3,000 nt)

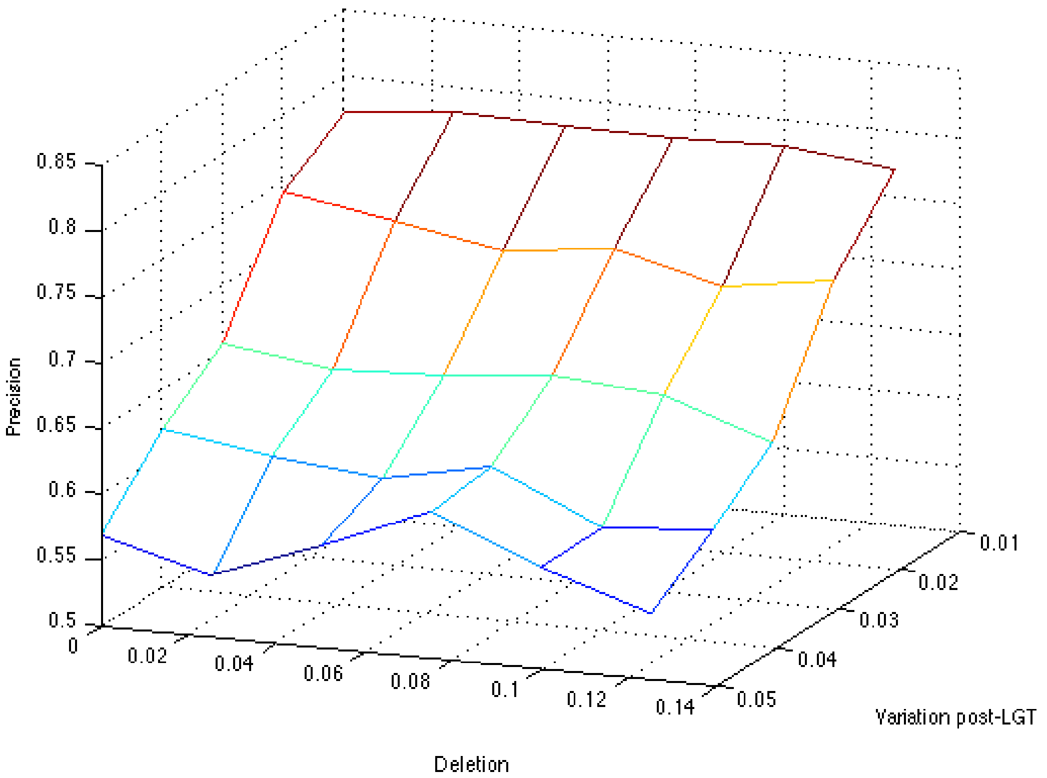

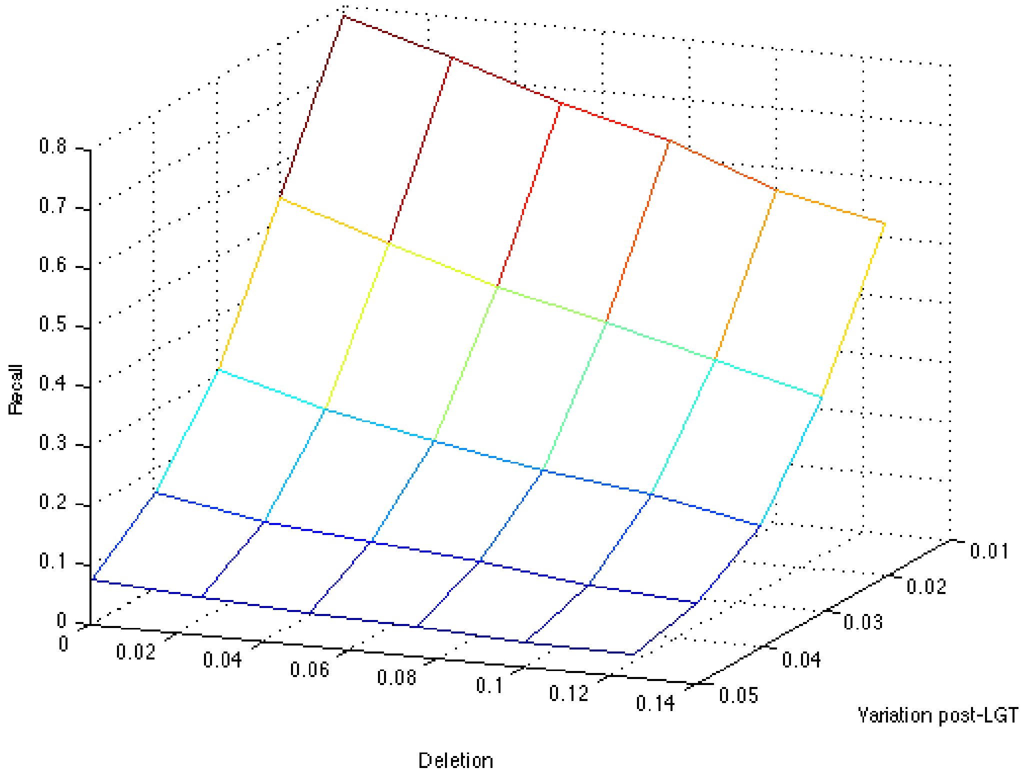

Performance on variation post-LGT and deletion (sequence length = 1,000 nt)

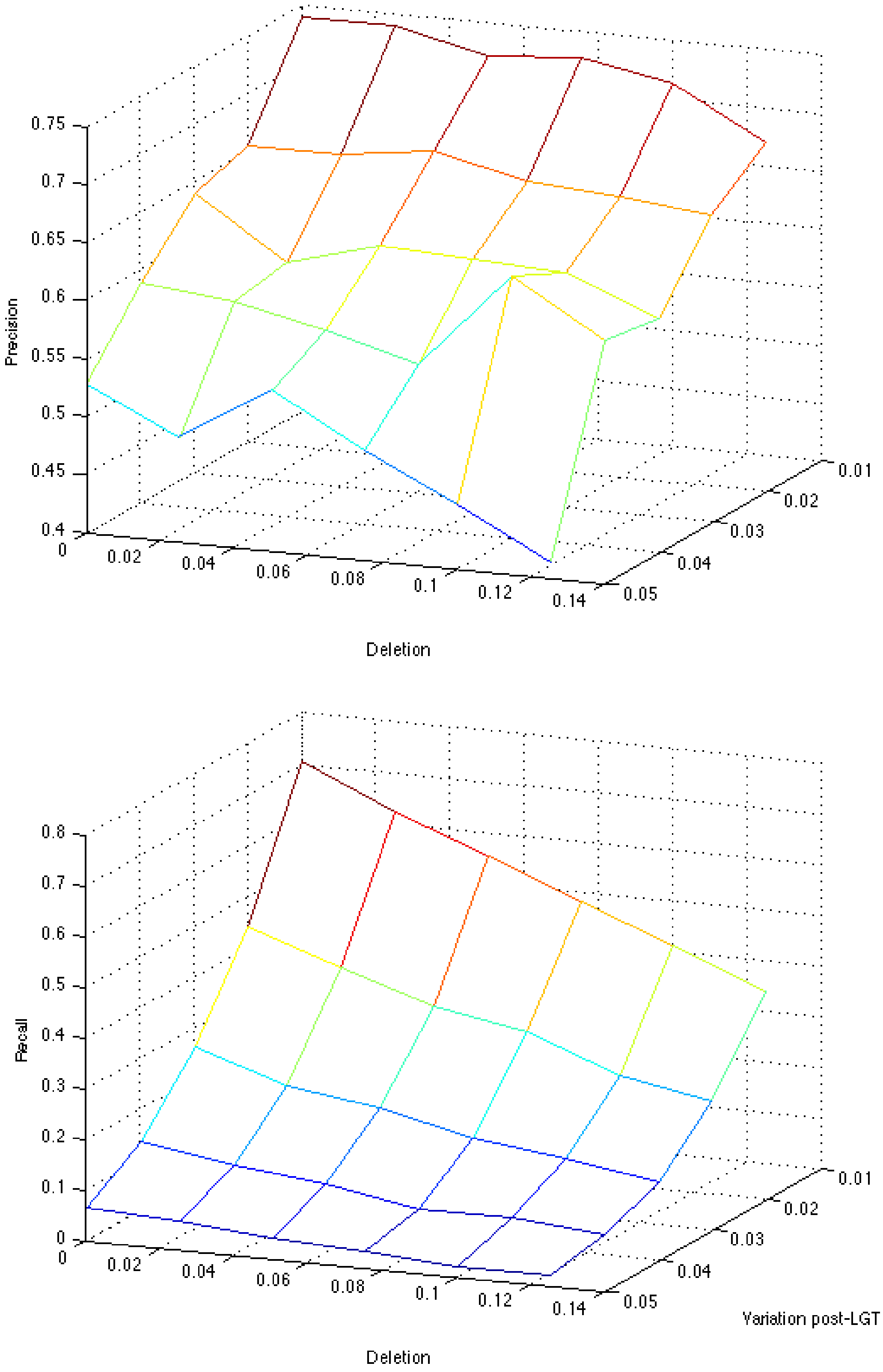

7 Supplementary Table S1. Detection of lateral regions in *Staphylococcus aureus* TW20 by TF-IDF, at *k* = 30 and *k* = 40

Annotated functions of proteins fully or partially contained within an LGT region of *Staphylococcus aureus* TW20, as discovered in this dataset by TF-IDF (*k* = 40). The first row is the length range of LGT segments selected for analysis.

		2000-3999 nt	4000-5999nt	6000+ nt	2000+ nt	2000+ nt
Annotated function[1]	Annotated in genome	Number	Number	Number	Number	%
adhesion / adhesion	4	0	0	2	2	50
antiporter	10	1	0	1	2	20
capsular polysaccharide	16	0	0	16	16	100
capsule	3	0	0	3	3	100
coagulase	1	0	0	1	1	100
efflux	4	1	0	1	2	50
integrase	9	0	1	0	1	11
lactamase	8	0	0	5	5	62
lysine	13	0	0	1	1	8
metalloproteinase /metallopeptidase	3	1	0	1	2	66
penicillin	6	1	0	3	4	66
permease	45	8	5	18	31	69
phage	249	12	14	0	26	11
recombinase	6	0	0	0	0	0
resistance protein	9	0	1	1	2	22
restriction	9	0	0	3	3	33
siderophore	5	0	0	5	5	100
surface protein	5	1	1	4	6	100
toxin	19	1	0	0	1	5
transport protein	28	3	1	7	11	39
transporter	98	14	3	28	45	46
transposase	30	0	2	2	4	13
uptake	4	1	1	0	2	50
ribosomal protein	60	2	1	5	8	13
polymerase (DNA/RNA)	15	5	2	1	8	53

Annotated functions of proteins fully or partially contained within an LGT region of *Staphylococcus aureus* TW20, as discovered in this dataset by TF-IDF (k = 30). The first row is the length range of LGT segments selected for analysis.

		2000-3999 nt	4000-6499 nt	6500+ nt	2000+ nt	2000+ nt
Annotated function[1]	Annotated in genome	Number	Number	Number	Number	%
adhesion / adhesion	4	0	0	4	4	100
antiporter	10	7	0	1	8	80
capsular polysaccharide	16	0	0	16	16	100
capsule	3	0	0	3	3	100
coagulase	1	0	0	1	1	100
efflux	4	1	1	1	3	75
integrase	9	2	1	0	3	33
lactamase	8	1	0	4	5	62
lysine	13	2	0	3	5	38
metalloproteinase /metallopeptidase	3	2	0	1	3	100
penicillin	6	3	2	2	(7)[2]	100
permease	45	11	7	15	33	73
phage	249	13	17	0	30	12
recombinase	6	2	0	0	2	33
resistance protein	9	1	0	1	2	22
restriction	9	0	0	3	3	33
siderophore	5	0	0	5	5	100
surface protein	5	0	1	2	3	60
toxin	19	1	0	0	1	5
transport protein	28	8	1	11	20	71
transporter	98	26	11	22	59	60
transposase	30	4	0	2	6	20
uptake	4	1	1	1	3	75
ribosomal protein	60	13	9	32	54	90
polymerase (DNA/RNA)	15	5	2	5	12	80

Notes:
1. As annotated in GenBank NC_017331.1
2. The 5′ and 3′ ends of the penicillin binding protein 2B gene fall into different inferred LGT regions.
